# Social Change and the Health of Sexual Minority Individuals: Do the Effects of Minority Stress and Community Connectedness Vary by Age Cohort?

**DOI:** 10.1007/s10508-022-02288-6

**Published:** 2022-04-11

**Authors:** David M. Frost, Ilan H. Meyer, Andy Lin, Bianca D. M. Wilson, Marguerita Lightfoot, Stephen T. Russell, Phillip L. Hammack

**Affiliations:** 1 Social Research Institute, University College London, 27-28 Woburn Square, London, WC1H 0AA UK; 2 The Williams Institute, University of California, Los Angeles, Los Angeles, CA USA; 3 School of Medicine, University of California, San Francisco, San Francisco, CA USA; 4 Department of Human Development and Family Sciences, University of Texas at Austin, Austin, TX USA; 5 Psychology Department, University of California, Santa Cruz, Santa Cruz, CA USA

**Keywords:** Discrimination, Stigma, Mental health, Physical health, Social well-being, Sexual orientation, Life course

## Abstract

This study examined the extent to which social stress stemming from a stigmatized social status (i.e., minority stress) was associated with three domains of health in younger as compared with older age cohorts of sexual minority individuals. Data were analyzed from the Generations Study, a longitudinal study using a probability sample (*N* = 1518) of age cohorts of sexual minority individuals in the USA. Exposure to a variety of minority stressors was associated with poorer health for all age cohorts. We hypothesized that because of improved social and legal environments in recent years, the associations between minority stress and health would be diminished in the younger cohort. As expected, we found that the associations between some minority stressors and health outcomes were diminished in the younger cohort compared to older cohorts. Positive associations between community connectedness and mental health and social well-being were observed for all participants but were attenuated in the younger cohort. Findings demonstrate the continuing negative association between minority stress and health among sexual minorities, which, despite some attenuation, persists even for young cohorts of sexual minority individuals in a more equal and accepting social climate.

**Supplementary Information:**

The online version contains supplementary material available at 10.1007/s10508-022-02288-6.

## Introduction

During the past two decades, research has consistently demonstrated poorer mental and physical health outcomes in sexual minority populations (i.e., lesbian, gay, bisexual, queer, and other individuals who do not identify as heterosexual) relative to their heterosexual cisgender peers (Lick et al., 2013; National Academies of Sciences, Engineering, & Medicine, 2020; Russell & Fish, 2016). These health disparities are theorized to be a result of the persistent stigmatization of sexual minority populations in most societies across the globe (Hatzenbuehler, 2017; Meyer, 2003, 2016). Although sexual minority health inequalities persist, the social climate for sexual minority individuals in Western contexts has become more accepting and equal (Meyer, 2016; Roberts, 2019). As a result of these social changes, some social scientists have posited that current cohorts of sexual minorities are growing up in a climate characterized by less stigma and discrimination compared to previous cohorts (McCormack, 2013; Savin-Williams, 2016). However, despite recent social changes, notable sexual orientation-related health disparities persist, and sexual minorities still face stigma and social disadvantage (Meyer, 2016; Russell & Fish, 2019). In the present study, we used data from the Generations Study (Meyer et al., 2020)—a probability sample of three age cohorts of sexual minority individuals in the USA—to examine the extent to which minority stress was associated with health and well-being and the degree to which this association differed between younger and older cohorts of sexual minority individuals.

### Social Stress as an Explanation for Sexual Minority Health Disparities

Researchers working across the social and health sciences have explained sexual minority health disparities using social stress paradigms (Frost, 2017; Hatzenbuehler & Pachankis, 2016; Meyer, 2003; Meyer et al., 2008). These models recognize that sexual minority populations are exposed to excess social stress due to the stigmatized status assigned to their identities by society. They also may experience thwarted access to stress-ameliorating resources (coping, social support) compared to their heterosexual peers as a result of social exclusion and marginalization (Meyer et al., 2008). These social stress frameworks contend that excess exposure to social stress and limited access to stress-ameliorating resources put sexual minority individuals at heightened risk of negative health outcomes, which explains health inequalities based on sexual orientation (Institute of Medicine, 2011).

### The Minority Stress Framework

The minority stress framework (Meyer, 2003) describes multiple forms of social stressors that sexual minority individuals are potentially exposed to as a result of their stigmatized social status in the form of acute stressful life events stemming from prejudice, chronic everyday forms of discrimination, expectations of rejection, stigma concealment, and internalized stigma. These minority stressors are theorized to represent an excess stress burden, which is not experienced by heterosexuals. Minority stressors can be located in terms of their proximity to the self and can be categorized as distal and proximal (Meyer, 2003).

#### Distal Minority Stressors

Distal minority stressors in the form of prejudice events like victimization are motivated by prejudice against sexual minority individuals that exists in the larger society. These events typically take place during interpersonal interactions (e.g., with colleagues at work or strangers in public) and may sometimes be illegal depending on the social context in which they occur (e.g., hate crimes, housing discrimination). Prejudice events experienced by sexual minorities can also occur at home and be perpetrated by family members, particularly among sexual minority youth (Ryan et al., 2009). Prejudice events have been demonstrated to have a negative impact on sexual minority individuals’ health, beyond the impact of general life events that are not motivated by prejudice (e.g., Frost et al., 2015). Repeated or chronic devaluations also reflect distal forms of minority stress. Forms of everyday discrimination (e.g., microaggressions), which may manifest in harassment and other instances of devaluation, rejection, and disrespect (e.g., being treated with less courtesy in stores or restaurants), are stressful even if they are not major life events (Swim et al., 2009).

#### Proximal Minority Stressors

Proximal forms of minority stress originate from a devaluing society but invoke people’s perceptions and evaluations of their relationship to the social context. Felt stigma has the potential to impact sexual minority people even in the absence of actual experiences of victimization or discrimination, because sexual minority individuals may approach social interactions expecting to be treated negatively. Felt stigma may be the result of hypervigilance on the part of individuals due to their awareness of their stigmatized status in society and that commonly held stereotypes and prejudice exist about their minority group (Meyer, 2003). Felt stigma constitutes minority stress due to the cognitive burden and anticipatory stress inherent to expectations of rejection. Related to expectations of rejection is stress associated with managing the visibility and concealment of one’s sexual orientation (Pachankis, 2007). Concealment can be thought of as a double-edged sword in that although keeping one’s sexual or gender minority identity a secret can be a shield from overt forms of minority stress (Rosario et al., 2001; Swank et al., 2013), concealing requires a significant cognitive effort on the part of the individual, which is demanding and stressful (Pachankis, 2007). Internalized stigma (also called internalized homophobia) refers to the self-application of negative social attitudes prevalent in society about sexual minorities (Frost & Meyer, 2009; Herek, 2009). In some of its most dangerous forms, it can lead to the condemnation of a person’s sexual minority identity. Internalized stigma is also characterized by an internal and psychological discrepancy between experiences of same-sex attraction and sexual desire and feelings that one should be heterosexual to conform to heteronormative expectations imposed by society (Herek, 2004, 2007).

#### Minority Stress and Sexual Minority Health

Studies that have utilized the minority stress framework have produced a strong body of evidence that exposure to minority stressors is associated with mental health problems, including DSM-diagnosable mood and anxiety disorders, symptoms of depression, substance use, and suicide ideation, as well as lower levels of psychological and social well-being (for reviews, see Hatzenbuehler & Pachankis, 2016; Meyer & Frost, 2013; Pitoňák, 2017). Exposure to minority stress has also been shown to be associated with increased physical health problems (Frost et al., 2015). Thus, minority stress has been hypothesized to be an explanation for elevated rates of mental and physical health outcomes and health risk behaviors observed in sexual minority populations (Lick et al., 2013).

#### The Role of Community Connectedness

In addition to articulating the pathways through which stigma constitutes minority stress and affects the health and well-being of sexual minorities, the minority stress framework (Meyer, 2003) identifies important resilience resources that can serve to reduce the impact of minority stress on health. Meyer (Frost & Meyer, 2012; Meyer, 2003) contended that a feeling of connection to a community of other sexual minority individuals (e.g., local LGBT community) represents a minority-specific coping resource that can provide a stress-ameliorating function for sexual minority individuals. Unlike individual coping strategies and social support—which are resilience resources available in varying degrees to all individuals regardless of sexual orientation—community connectedness is a unique resource available to sexual minority individuals and is therefore thought to be especially relevant in reducing the effects of stressors unique to the sexual minority experience.

In line with recent attempts to integrate social stress and resilience perspectives (e.g., Fredriksen-Goldsen et al., 2014; Frost, 2017; Meyer, 2015; Perrin et al., 2020), the present study considers both the stress-ameliorating role that community connectedness plays as a community coping resource, along with the potential direct salutogenic effect that feeling connected to a community of other sexual minorities may serve for the health of sexual minority individuals. In the general population, the fundamental human need to belong is associated with positive individual and social outcomes (Baumeister & Leary, 1995). Among sexual minorities specifically, positive effects of community connectedness have been demonstrated in various studies regarding health outcomes, including mental health, social and psychological well-being, and physical health risk behaviors (e.g., Kertzner et al., 2009; Perrin et al., 2020; Scroggs & Vennum, 2021).

### Social Change and Minority Stress

The last two decades have witnessed significant increases in positive attitudes toward equality and social inclusion of sexual minorities in the USA. Attitudes toward sexual minority individuals and same-sex couples have drastically improved, with most of the US population supporting same-sex marriage for the first time as of 2014 (Fingerhut, 2016). Acceptance of and positive attitudes toward sexual minorities is very strongly related to age, with younger people having more favorable attitudes than older people (Fingerhut, 2016). As a result, younger sexual minority people have experienced peers who are vastly more accepting than did older sexual minority people. Improving attitudes toward same-sex sexuality and relationships among younger cohorts have led some researchers to contend that sexual minorities now come of age in a “post-gay” era (e.g., McCormack, 2013; Savin-Williams, 2016). This work suggests that sexual minority emerging adults are not as marginalized and stigmatized as older cohorts have been and thus they should have fewer experiences of minority stress, resulting in better health when compared with older sexual minority cohorts.

Research evidence does not directly support this hypothesis. Sexual minority emerging adults continue to experience minority stress in the form of victimization, bullying, and marginalization (e.g., Baams et al., 2015; Frost et al., 2019; Meyer et al., 2021) and continue to experience health disparities (Meyer, 2016; Russell & Fish, 2016). Thus, it is possible that despite improved social conditions, sexual minority emerging adults continue to experience stigma and victimization. If so, they need to navigate a new, liberating narrative of normality regarding same-sex desire alongside experiencing continued stigmatized status in the course of their development (e.g., Cohler & Hammack, 2007; Hammack et al., 2009).

### A Life Course Perspective

A life course perspective (e.g., Elder, 1998) is needed to investigate the role of social change in the experiences of minority stress and community connectedness and their resulting implications for health and well-being for sexual minority populations (Hammack et al., 2018; Institute of Medicine, 2011). The concept of cohort has historically been useful in the study of social change. We define cohort using Ryder’s (1965) classic definition as “the aggregate of individuals (within some population definition) who experience the same event within the same time interval” (p. 845).

Cohort defining events reflecting social change surrounding the acceptance of sexual minority individuals in the USA include most notably the decriminalization of homosexuality (Lawrence v. Texas) in 2003, the repeal of the ban on serving openly in the military (“Don’t ask, don’t tell”) in 2010, and the Supreme Court decision to legalize same-sex marriage (Obergefell v. Hodges) in 2015. Sexual minority people who experienced these events during periods critical to the development of sexuality and sexual identities (i.e., puberty and adolescence) can be considered to be a cohort meaningfully distinct from previous cohorts that did not experience similar events during similar developmental periods (Hammack et al., 2018; Meyer et al., 2021). Sexual minority emerging adults will have experienced childhood and adolescence when society changed in ways that made it possible for them to imagine futures in which they were able to participate in society in ways that were restricted for previous cohorts (e.g., marriage, adoption, workplace inclusion). The extent to which this movement toward a more equal and accepting social environment for sexual minorities translates to a diminished association between minority stress and health has yet to be examined. Further, the extent to which a more accepting general social climate translates to a decreased reliance on sexual minority communities for health-promoting functions of support and affirmation has also yet to be examined.

Our study focused on assessing whether positive social changes have implications for the association between minority stress and health. Namely, is minority stress associated with the health of sexual minority emerging adults (i.e., younger cohorts) in the same way as it has been for older sexual minority cohorts? Research on the association between minority stress and health has not adequately assessed the role of the shifting sociohistorical context. If new cohorts of sexual minority emerging adults are indeed experiencing more affirming and inclusive social climates compared to previous cohorts, we would expect that minority stress would be less strongly associated with their health. Similarly, community connectedness, as an LGBT-specific group-level resilience resource, may be less likely to serve a stress-buffering function for younger cohorts of sexual minority people as connections to communities of other sexual minorities would be less important than they were to previous cohorts. A more accepting social climate means that there is less of a need for a community support and affirmation. If the stigma surrounding sexual minority identities is less defining and less central to the lived experiences of young sexual minority people today, then they may require less resilience and coping when compared with older sexual minorities.

Our study fills a gap in the literature. Other studies focused on the experiences of sexual minority adolescents and emerging adults—who are theorized to experience improved social climates—demonstrated associations between experiences of minority stress and poorer health and well-being, but lacked age cohort comparisons to directly explore these issues. Although some studies have investigated age cohort differences in associations between minority stress and health and well-being (e.g., Vale & Bisconti, 2021), they have largely focused on older cohorts, were based on cross-sectional data, lacked representative samples, investigated only a few minority stressors, and were limited to single indicators of health.

### The Current Study: Aims and Hypotheses

Using a life course perspective, we assessed the role of social change in the relationship between minority stress, community connectedness, and health. We compared the experiences of a cohort of young sexual minority adults (ages 18–25)—who came of age in a social climate characterized by events reflecting shifts toward greater social acceptance and equality—with older cohorts of sexual minorities.

**Fig. 1:**
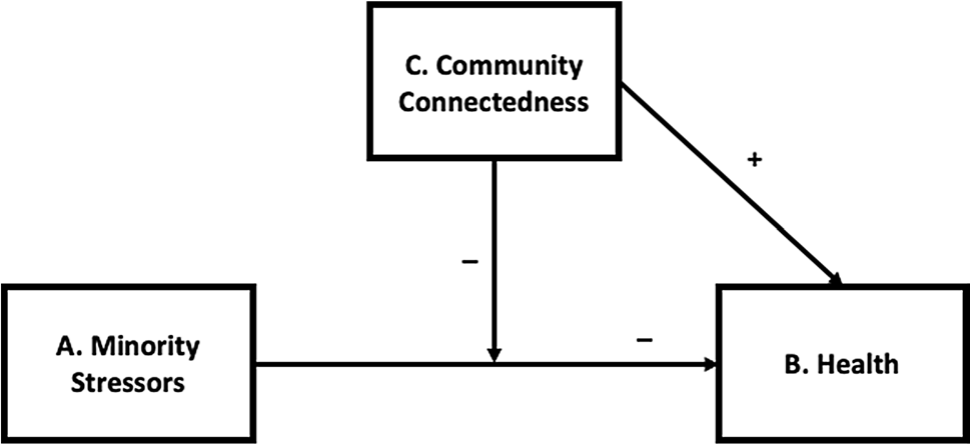
Hypothesized relationships between minority stressors, community connectedness, and health

We analyzed three waves of data from the Generations Study (Meyer, 2020) to examine whether foundational principles of the minority stress model (Fig. 1; Meyer, 2003) held true for a cohort of young sexual minority adults (aged 18–25 at baseline) as compared with older cohorts of sexual minority individuals included in the Generations Study. Following general models of social stress and health, the associations between social stress, resilience, and health are theorized not to be specific to any given disorder or condition and are intended to be extended to health as a collective domain more generally (Meyer, 2010). To provide a robust test of our hypotheses, we assess the associations of minority stressors (including victimization, everyday discrimination, felt stigma, concealing, and internalized stigma) with three domains of health outcomes. We included previously validated measures of mental health in the form of nonspecific psychological distress and physical health in the form of self-reported health. When conceptualizing domains of mental health, researchers have called for a shift away from focusing exclusively on the presence or absence of pathology in the form of disorder or symptoms of disorder, and instead broaden the focus to include positive indicators of mental health in the form of well-being (e.g., Fredriksen-Goldsen et al., 2014; Kertzner et al., 2009). Thus, we also included social well-being (Keyes, 1998) as a third domain of health (World Health Organization, n.d.).

We hypothesized that (1) members of the younger cohort would experience better health than their peers in older cohorts due to the improved social environment in which they grew up; (2) exposure to minority stressors would be associated with poorer health (Fig. 1, A → B); (3) this association would be weaker for younger than older sexual minority individuals as a result of their experiences coming of age in a more positive and accepting social and policy climate; and (4) community connectedness would play a salutogenic role in its relationship to health (Fig. 1, C → B), which would be weaker for younger cohorts than older cohorts, given it has been suggested that a connection to sexual minority communities is less important for younger sexual minorities. Finally, we hypothesized that community connectedness would play an additional stress-buffering role (Fig. 1, [A × C] → B), reducing the negative association of minority stress with health, which would be weaker for younger sexual minority individuals compared to their peers in older cohorts.

## Method

The current study analyzed publicly available data from the Generations Study (Meyer, 2020).

### Participants and Procedure

A detailed account of the probability sampling methods used in the Generations Study have been published (Meyer et al., 2020). Participants were screened and recruited using the Gallup Daily Tracking Survey: A telephone interview of a national probability sample of 1,000 adults aged 18 or older daily (350 days a year) using a dual-frame sampling procedure, which includes random-digit dialing to reach both landline and cellphone users, as well as an additional random selection method for choosing respondents in households reached through landlines.

Generations participants were screened and enrolled in the study between March 28, 2016, and March 30, 2017. An enhancement oversample, recruiting Black and Latino respondents, was screened and enrolled between April 1, 2017, and March 30, 2018. The Generations Study used a two-phase recruitment procedure. In the first phase, all sexual minority individuals were identified by a question asked of all Gallup respondents: Do you, personally, identify as lesbian, gay, bisexual, or transgender (LGBT)? In the second phase, respondents who identified as LGBT were then assessed for sexual identity, gender identity, and other eligibility criteria and if eligible, invited to participate in the Generations study and sent a survey questionnaire by mail or email link.

Respondents were eligible if they identified as sexual minority (and not transgender); were in the age groups targeted for the three cohorts under investigation in Generations (18–25, 34–41, or 52–59); were Black, Latino, or White, or multiracial including one of the these race/ethnic identities; completed sixth grade; and spoke English well enough to conduct the phone interview in English. (Respondents who were transgender, regardless of their sexual orientation, were screened for participation in a parallel study; respondents who identified as nonbinary but not transgender were included in the Generations Study.)

Respondents who were eligible and agreed to participate were emailed or mailed a survey questionnaire to complete by self-administration (via a web link or printed questionnaire, respectively). Respondents received $25 (an Amazon gift card by email or cash by mail). Following this baseline survey, respondents were asked to complete two follow-up surveys using the same modality (mail or web) and received the same compensation of $25 per interview, one year apart, at Year 2 and Year 3. The study protocol was reviewed and approved by the institutional review board at University of California, Los Angeles.

A detailed description of the Generations sample (*N* = 1518) has been published elsewhere (Meyer et al., 2020). The majority of participants identified as lesbian or gay (46.29%), 40.02% identified as bisexual, and 13.70% identified with other sexual orientation labels (e.g., pansexual, queer). The younger generation constituted 44.14% of the sample, and the middle and older cohorts together constituted 55.86%. The sample was diverse in term of gender (55.01% cisgender female, 37.58% cisgender male, 7.41% nonbinary or genderqueer) and race and ethnicity (62.66% White, 16.52% Black or African American, 21.26% Latino or Hispanic). In terms of socioeconomic status, 42.51% of participants had a high school education or less and 8.30% were unemployed. Approximately one quarter (25.95%) of participants lived 60 miles or more from an LGBT community center.

Of the original sample, 1331 participants were recontacted for follow-up. A total of 707 (53.12%) participants were retained from Wave 1 to Wave 3, and 616 (46.28%) participants completed all three waves of the survey. There were some expected differences observed between those who were retained versus those who were not (Krueger et al., 2020). Specifically, members of the younger cohort were more likely to have been lost to follow up. In addition, some differences were seen with race, wherein Black and African American and Latino participants were more likely to have been lost to follow up than White participants. Additionally, respondents who lived 60 miles or more from the nearest LGBT health center were more likely to have been lost to follow-up from Wave 1 to Wave 3.

### Measures

The following measures were included in each wave of the survey.

#### Health Outcomes

##### Mental Health

Mental health was measured using the six-item K6 scale (Kessler et al., 2002), which measures nonspecific psychological distress during the past 30 days in terms of the frequency (0 = none of the time to 4 = all of the time) of participants feeling the following symptoms: (a) nervous, (b) hopeless, (c) restless or fidgety, (d) so depressed that nothing could cheer them up, (e) that everything was an effort, and (f) worthless. Scale values were calculated by summing the items and ranged from 0 to 24, with higher values indicating more distress (α_w1_ = 0.88, α_w2_ = 0.89, α_w3_ = 0.89).

##### Physical Health

Physical health was measured using the single-item General Health Rating from the SF-12 (Ware et al., 1996): “In general, would you say your health is…” Participants responded on a 5-point scale ranging from excellent to poor, with higher scores indicating worse self-rated health. Ratings were provided of participants’ health at the time of participation. This approach to measuring self-appraised physical health has demonstrated validity regarding morbidity and mortality outcomes (Idler & Benyamini, 1997).

##### Social Well-Being

Social well-being was assessed using Keyes’ (1998) Social Well-Being scale, which consists of 15 items, including: “The world is becoming a better place for everyone” and “I have something valuable to give to the world.” Each item was rated on a 7-point Likert scale ranging from 1 = strongly disagree to 7 = strongly agree. Responses were provided based on participants’ level of agreement at the time of participation. Scale values were calculated as the mean of the items and ranged from 1 to 7, with higher values representing greater social well-being (α_w1_ = 0.81, α_w2_ = 0.81, α_w3_ = 0.83).

#### Minority Stressors

##### Victimization

A six-item measure (Herek, 2009) was used to assess the frequency of victimization experienced since age 18 at baseline and in the past year at each follow-up wave. Items included being hit, beaten, physically attacked, or sexually assaulted; and someone threw an object at you. Participants were asked to report how often they experienced any of these forms of victimization and were not asked to limit their reporting to victimization related to sexual orientation. Responses were provided on scale of 1 = never to 4 = three or more times. Scale values were calculated as a mean of the items (*α*_*w*1_ = 0.80, *α*_*w*2_ = 0.66, *α*_*w*3_ = 0.72).

##### Everyday Discrimination

Williams et al.’s (1997) Everyday Discrimination Scale was used to assess chronic experiences of discrimination or unfair treatment during the past year. Example items included: “you were treated with less courtesy than other people,” “you were treated with less respect than other people,” and “you were called names or insulted.” Participants were asked to report how often they experienced any of these forms of discrimination and were not asked to limit their reporting to discrimination related to sexual orientation. Responses were recorded on a 4-point Likert scale ranging from never to often. Scale values were calculated as the mean of the items and ranged from 1 to 4, with higher values representing more everyday discrimination (*α*_*w*1_ = 0.91, *α*_*w*2_ = 0.91, *α*_*w*3_ = 0.91).

##### Felt Stigma

The Felt Stigma Scale (Herek, 2009) assessed respondents’ awareness and experiences of minority stress related to expectations of rejection and devaluation. Example items were: “most people where I live think less of a person who is LGB,” “most employers where I live will hire openly LGB people if they are qualified for the job,” and “most people where I live would not want someone who is openly LGB to take care of their children.” Responses were recorded on a 5-point Likert scale ranging from strongly disagree to strongly agree and not limited to a specific timeframe. Scale values were calculated as the mean of the items and ranged from 1 to 5, with higher values representing greater felt stigma (*α*_*w*1_ = 0.70, *α*_*w*2_ = 0.74, *α*_*w*3_ = 0.78).

##### Concealment

Concealment of sexual orientation was measured following the approach used in a previous study using probability sampling (Meyer et al., 2002). Specifically, respondents were asked the degree of disclosure of sexual orientation to (a) family, (b) straight friends, (c) co-workers, and (d) health care providers. Participants described the extent to which they were out of the closet to each group on a scale of 1 = out to none to 4 = out to all and not limited to a specific timeframe. Scores were reverse coded to reflect concealment (as opposed to outness) so that higher scores reflected greater concealment. In the current study, we used only one item reflecting concealment from family, because concealment from family is a more stringent indicator of concealment given sexual minorities often “come out” to friends and peers before family (Grierson & Smith, 2005; Riley, 2010). Additionally, because researchers have highlighted the importance of familial acceptance (i.e., parental approval) for health among sexual minority populations (Ryan et al., 2009, 2010), stigma concealment in the family context was determined to be the most relevant indicator of concealment for the purposes of the current study. Additional indicators of concealment were determined to be less appropriate indicators of concealment given they may not apply to all participants’ experiences (e.g., participants who are students, unemployed, or retired do not have co-workers).

##### Internalized Stigma

Internalized stigma was assessed using the Internalized Homophobia scale (Herek et al., 2009). Example items are: “I have tried to stop being attracted to people who are the same sex as me,” “I wish I weren’t LGB,” and “I feel that being LGB is a personal shortcoming for me.” Responses were recorded on a 5-point Likert scale ranging from strongly disagree to strongly agree and not limited to a specific timeframe. Scale values were calculated as the mean of the items and ranged from 1 to 5, with higher values representing greater internalized stigma (*α*_*w*1_ = 0.75, *α*_*w*2_ = 0.78, *α*_*w*3_ = 0.76).

#### Community Connectedness

##### Connectedness to the LGBT Community

The Connectedness to the LGBT Community Scale (Frost & Meyer, 2012) was used assess the desire for and strength of affiliation with a community of other sexual minorities. Seven of the original eight items were used, omitting the last item, which referred only to a sense of connection with other people who share the same gender and sexual orientation as the participant, because the survey could not be personalized to each participant’s gender and sexual orientation. The scale was also adapted from the original focus on a geographically specific community (e.g., New York City’s LGBT community) to a general framing for the national survey. Items included: “you feel you’re a part of the LGBT community” and “you are proud of the LGBT community.” Responses were recorded on a 4-point Likert scale ranging from agree strongly to disagree strongly and not limited to a specific timeframe. Responses were reverse coded. Scale values were calculated as the mean of the items and ranged from 1 to 4, with higher scores representing greater community connectedness (*α*_*w*1_ = 0.86, *α*_*w*2_ = 0.86, *α*_*w*3_ = 0.87).

### Analytic Approach

Tests of the social change hypotheses centered on predictions that the experiences of the younger cohort of sexual minority emerging adults would be different than older cohorts of sexual minority individuals who came of age in less positive and accepting social climates. Specifically, members of the middle and older cohorts included in the Generations Study were considered to have come of age in social contexts that shared similarities surrounding the status of sexual minority identities, same-sex behavior and relationships that were not experienced by the younger cohort. For example, the middle and older cohorts’ experiences of puberty and adolescence were similar in that they were characterized by social contexts in which the majority of the US population “disapproved” of same-sex marriage and homosexual behavior, homosexual behavior was against the law, and same-sex marriage was not legal. The younger cohort of sexual minority emerging adults’ experiences were markedly different from both the middle and older cohorts in the Generations Study, given they were characterized by a constellation of cohort-defining events reflecting more social acceptance and equality, as described in the introduction. Following this conceptual justification based in life course research and the use of cohort comparison to examine social change, we used a dichotomous variable for cohort that compared the younger cohort (coded as 1) to the middle and older cohorts combined (coded as 0). Time was coded as: first wave = 0, one-year follow-up = 1, and two-year follow-up = 2. All analyses were adjusted for sex assigned at birth (female = 1, male = 0), gender identity (nonbinary = 1, cisgender = 0); sexual identity (plurisexual [e.g., pansexual, bisexual, queer] = 1, lesbian or gay = 0); education level (1 = high school or less, 0 = some college or greater); and race and ethnicity (Black or Latinx separately; yes = 1, no = 0), with White as the referent group.

To test the hypothesized relationships depicted in Fig. 1, we utilized generalized estimating equations (GEEs). Using GEEs allowed us to test a prediction model that yields group-level, rather than person-level, estimates of effects (Ballinger, 2004; Hubbard et al., 2010). GEEs were also chosen given their ability to handle longitudinal data with missing waves within cases. Specifically, GEEs retain all possible information from participants, dropping only missing waves within a participant’s data. GEEs do not require dropping the entirety of a participant’s data if they did not participate in all waves (case-wise deletion), and GEEs do not require imputation of missing data for missing waves (Twisk & de Vente, 2002). Thus, we used GEEs to estimate models separately for each health outcome, which included: (a) minority stressors and community connectedness as predictor variables; (b) interactions between cohort and minority stressors and community connectedness to examine whether cohorts differed in the associations between minority stress, community connectedness, and health; and (c) interactions between community connectedness and minority stressors to test whether community connectedness moderated the association between minority stress on health. We also tested models involving three-way interactions among cohort, connectedness, and minority stressors, but none of these interactions were substantial or statistically significant, and the results of these analyses are not reported.

Missing data for predictors and outcomes were minimal (i.e., ≤ 4.1%) and were imputed only if other variables were measured for the participant in the same wave. To impute missing values, we used a single imputation by chained equations (fully conditional specification), using predictive mean matching (Little, 1988; Morris et al., 2014). Contemporaneous longitudinal associations between predictor variables and outcomes are reported. Continuous variables were grand-mean-centered to address issues related to multicollinearity and interpretability for interaction terms.

## Results

### Descriptive Statistics

Means and SDs for minority stress variables and community connectedness are presented separately for the younger and older cohorts in Table 1. Based on interpretation of effect size (i.e., Cohen’s d), we observed a large magnitude of difference in concealment from family, with members of the younger cohort being more concealed than members of the older cohorts. We observed a medium magnitude mean difference with regard to everyday discrimination, wherein members of the younger cohort reported higher levels than members of the older cohorts. We observed a medium magnitude difference between the cohorts on lifetime victimization, with members of the older cohort reporting more lifetime victimization than members of the younger cohort. A medium magnitude difference was observed with younger cohort members reporting higher levels of connectedness than older cohorts. Small magnitude mean differences between the cohorts were observed in internalized stigma, with younger cohorts reporting higher levels than older cohorts. Negligible differences in felt stigma were observed with confidence intervals spanning zero.

**Table 1:** Means and SDs for minority stress and connectedness variables by cohort

Variable	Younger cohort		Older cohorts		Cohen's *d*	95% Confidence interval	
	M	SD	M	SD		Lower	Upper
Victimization	1.80	0.77	2.10	0.83	0.37	0.27	0.48
Discrimination	2.09	0.71	1.76	0.64	− 0.49	− 0.59	− 0.39
Felt Stigma	2.66	0.95	2.66	0.94	0.00	− 0.10	0.10
Concealment From Family	1.49	1.04	0.74	0.98	− 0.75	− 0.85	− 0.64
Internalized Stigma	1.71	0.77	1.56	0.74	− 0.19	− 0.30	− 0.09
Connectedness	3.03	0.56	2.91	0.56	− 0.21	− 0.31	− 0.11

Means and SDs are presented for Wave 1 data only

Results of tests of the hypothesized relationships specified in Fig. 1 are presented separately for each health outcome.

### Outcome 1: Psychological Distress

As shown in Table 2, Model 1, members of the younger cohort reported moderately higher levels of psychological distress than members of the middle and older cohort combined, and levels of psychological distress demonstrated small but appreciable increases over time, on average.

**Table 2:** Generalized estimating equations predicting psychological distress based on minority stress and community connectedness across cohort and time

Parameter	Model 1: Main effects				Model 2: Cohort by minority stress interactions				Model 3: Connectedness by minority stress interactions			
	B	95% CI		*p*	B	95% CI		*p*	B	95% CI		*p*
		Lower	Upper			Lower	Upper			Lower	Upper	
Intercept	5.51	5.11	5.90	< 0.01	5.50	5.10	5.90	< 0.01	5.52	5.12	5.91	< 0.01
Black	− 0.86	− 1.51	− 0.21	0.01	− 0.85	− 1.49	− 0.20	0.01	− 0.85	− 1.49	− 0.20	0.01
Latino	0.04	− 0.52	0.59	0.90	0.04	− 0.52	0.59	0.90	0.04	− 0.51	0.60	0.88
Female Sex	0.21	− 0.24	0.66	0.37	0.19	− 0.26	0.63	0.41	0.18	− 0.27	0.63	0.43
Non-Binary Gender	1.17	0.20	2.15	0.02	1.14	0.17	2.11	0.02	1.15	0.18	2.11	0.02
Plurisexual	1.28	0.72	1.84	< 0.01	1.29	0.73	1.85	< 0.01	1.30	0.75	1.86	< 0.01
Highschool or Less	0.51	− 0.07	1.09	0.09	0.51	− 0.07	1.10	0.09	0.51	− 0.08	1.09	0.09
Younger Cohort	2.21	1.70	2.72	< 0.01	2.18	1.67	2.70	< 0.01	2.20	1.68	2.72	< 0.01
Time	0.32	0.14	0.51	< 0.01	0.30	0.12	0.49	< 0.01	0.32	0.13	0.51	< 0.01
Victimization	0.67	0.39	0.96	< 0.01	0.60	0.28	0.93	< 0.01	0.67	0.39	0.96	< 0.01
Discrimination	2.64	2.29	2.99	< 0.01	2.74	2.27	3.20	< 0.01	2.64	2.29	2.99	< 0.01
Felt Stigma	0.46	0.23	0.69	< 0.01	0.69	0.38	1.01	< 0.01	0.46	0.23	0.69	< 0.01
Concealment From Family	0.14	− 0.10	0.38	0.24	0.08	− 0.22	0.38	0.61	0.15	− 0.08	0.39	0.20
Internalized Stigma	0.39	0.10	0.69	0.01	0.34	− 0.04	0.71	0.08	0.39	0.09	0.68	0.01
Connectedness	− 0.42	− 0.80	− 0.05	0.03	− 0.75	− 1.21	− 0.29	< 0.01	− 0.40	− 0.77	− 0.03	0.03
Younger * Victimization					0.22	− 0.35	0.78	0.45				
Younger * Discrimination					− 0.17	− 0.85	0.51	0.62				
Younger * Stigma					− 0.60	− 1.05	− 0.15	0.01				
Younger * Concealment					0.09	− 0.33	0.52	0.66				
Younger * Internalized					0.11	− 0.47	0.68	0.72				
Younger * Connectedness					0.82	0.07	1.58	0.03				
Connectedness * Victimization									0.03	− 0.47	0.52	0.92
Connectedness * Discrimination									− 0.01	− 0.60	0.59	0.98
Connectedness * Stigma									− 0.19	− 0.56	0.19	0.33
Connectedness * Concealment									0.28	− 0.07	0.63	0.12
Connectedness * Internalized									− 0.08	− 0.55	0.39	0.75
Scale/Link Parameter	18.58				18.49				18.58			

Higher levels of victimization, everyday discrimination, felt stigma, and internalized stigma, were associated with elevated levels of psychological distress. The effect sizes corresponding to these associations were small to moderate, with the exception of everyday discrimination, which was associated with large increases in psychological distress. The association between concealment and psychological distress was negligible (CI spanning zero). Increased community connectedness was associated with small but appreciable decreases in psychological distress.

**Fig. 2:**
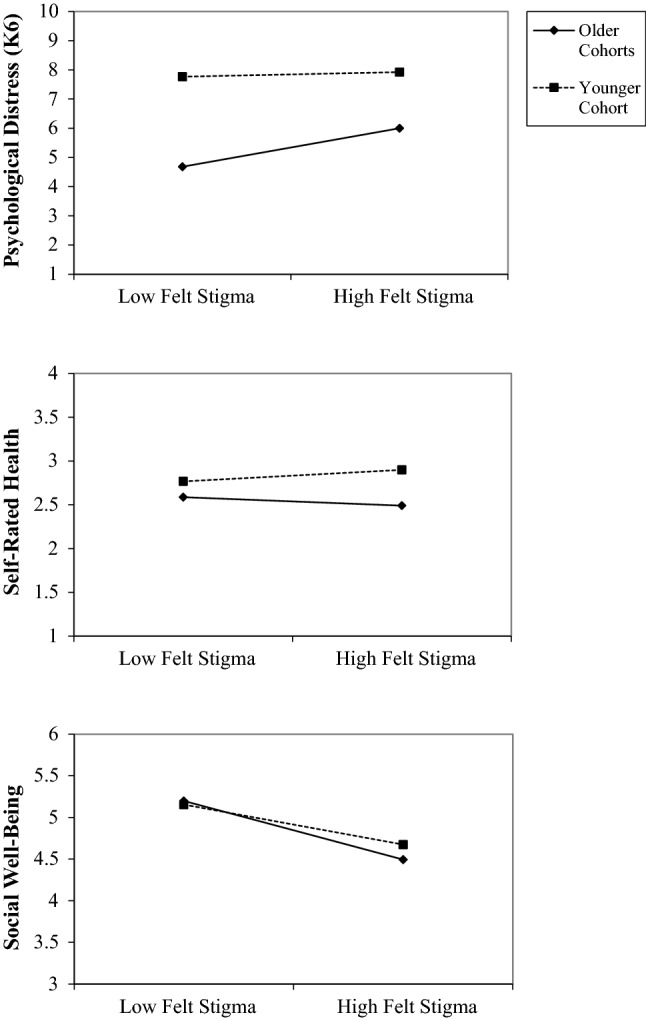
Age cohort as a moderator of the relationship between felt stigma and health outcomes

**Fig. 3:**
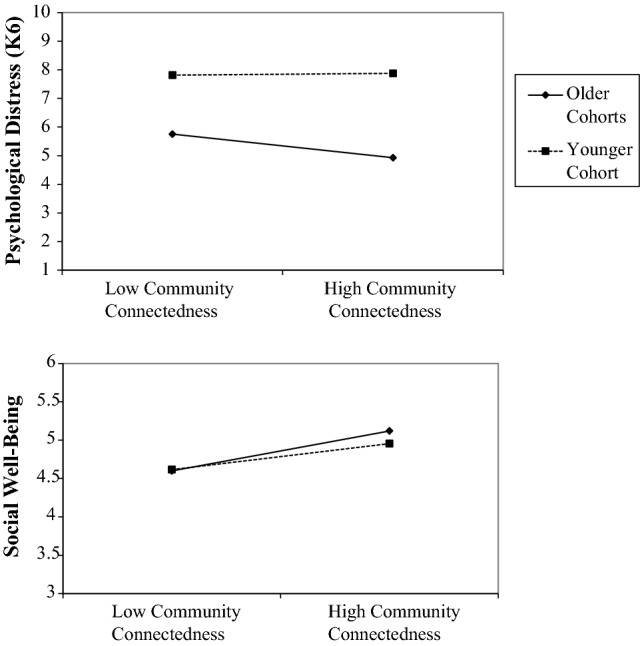
Age cohort as a moderator of the relationship between community connectedness and health outcomes

Model 2 (Table 2) presents tests of interactions examining the extent to which the associations between minority stress, community connectedness, and psychological distress varied by cohort. We observed an interaction between felt stigma and cohort (see Fig. 2), in which the association between stigma and psychological distress was more pronounced (small to moderate in magnitude) among members of the older cohorts than among members of the younger cohort (effect size near zero). Estimates of the interactions between cohort and the other minority stressors in models predicting psychological distress were near zero, suggesting negligible interactions. We observed an interaction between community connectedness and cohort in predicting psychological distress (see Fig. 3), in which the association between community connectedness and psychological distress was more pronounced (moderate effect size) for members of the older cohorts, but negligible (near zero) among members of the younger cohort.

Model 3 (Table 2) presents tests of interactions examining the extent to which the association between minority stress and psychological distress varied by levels of community connectedness. The effect sizes of the interactions between minority stressors and community connectedness in predicting psychological distress were negligible (CIs spanning zero).

### Outcome 2: Self-Rated Health

As shown in Table 3, Model 1, members of the younger cohort reported substantially higher levels of self-rated health (i.e., better health) than members of the older cohorts, and levels of self-rated health evidenced small decreases over time, on average, for all participants.

**Table 3:** Generalized estimating equations predicting self-rated health based on minority stress and community connectedness across cohort and time

Parameter	Model 1: Main effects				Model 2: Cohort by minority stress interactions				Model 3: Connectedness by minority stress interactions			
	B	95% CI		*p*	B	95% CI		*p*	B	95% CI		*p*
		Lower	Upper			Lower	Upper			Lower	Upper	
Intercept	2.53	2.44	2.62	< 0.01	2.54	2.44	2.63	< 0.01	2.52	2.43	2.61	< 0.01
Black	0.06	− 0.07	0.20	0.36	0.06	− 0.08	0.20	0.39	0.06	− 0.07	0.19	0.39
Latino	− 0.07	− 0.19	0.06	0.29	− 0.07	− 0.19	0.06	0.29	− 0.07	− 0.19	0.05	0.25
Female Sex	− 0.11	− 0.21	− 0.02	0.02	− 0.12	− 0.22	− 0.02	0.02	− 0.11	− 0.21	− 0.01	0.03
Non-Binary Gender	− 0.17	− 0.37	0.03	0.09	− 0.17	− 0.36	0.03	0.11	− 0.17	− 0.36	0.03	0.10
Plurisexual	− 0.16	− 0.28	− 0.05	0.01	− 0.16	− 0.28	− 0.04	0.01	− 0.17	− 0.28	− 0.05	0.01
Highschool or Less	− 0.16	− 0.27	− 0.04	0.01	− 0.16	− 0.28	− 0.04	0.01	− 0.15	− 0.27	− 0.03	0.01
Younger Cohort	0.30	0.19	0.40	< 0.01	0.30	0.20	0.40	< 0.01	0.30	0.20	0.41	< 0.01
Time	− 0.08	− 0.12	− 0.04	< 0.01	− 0.07	− 0.11	− 0.03	< 0.01	− 0.08	− 0.12	− 0.04	< 0.01
Victimization	− 0.09	− 0.15	− 0.03	0.01	− 0.08	− 0.15	0.00	0.04	− 0.09	− 0.15	− 0.02	0.01
Discrimination	− 0.31	− 0.38	− 0.23	< 0.01	− 0.32	− 0.43	− 0.22	< 0.01	− 0.31	− 0.38	− 0.23	< 0.01
Felt Stigma	− 0.16	− 0.20	− 0.11	< 0.01	− 0.21	− 0.27	− 0.14	< 0.01	− 0.16	− 0.21	− 0.11	< 0.01
Concealment From Family	− 0.03	− 0.08	0.02	0.29	0.00	− 0.07	0.07	0.94	− 0.03	− 0.08	0.02	0.19
Internalized Stigma	− 0.04	− 0.10	0.02	0.18	− 0.05	− 0.13	0.04	0.27	− 0.04	− 0.10	0.02	0.22
Connectedness	0.05	− 0.03	0.13	0.25	0.05	− 0.06	0.16	0.37	0.04	− 0.04	0.12	0.32
Younger * Victimization					− 0.03	− 0.15	0.08	0.60				
Younger * Discrimination					0.02	− 0.12	0.17	0.76				
Younger * Stigma					0.12	0.03	0.21	0.01				
Younger * Concealment					− 0.05	− 0.14	0.04	0.30				
Younger * Internalized					0.02	− 0.10	0.14	0.75				
Younger * Connectedness					− 0.02	− 0.18	0.13	0.78				
Connectedness * Victimization									− 0.02	− 0.12	0.08	0.73
Connectedness * Discrimination									0.11	− 0.01	0.23	0.07
Connectedness * Stigma									− 0.02	− 0.09	0.06	0.64
Connectedness * Concealment									− 0.09	− 0.16	− 0.02	0.01
Connectedness * Internalized									0.02	− 0.08	0.11	0.72
Scale/Link Parameter	0.86				0.85				0.85			

Regarding the associations between minority stressors, higher levels of victimization, everyday discrimination, and felt stigma, were associated with decreased levels of self-rated health. Similar to psychological distress, these associations were small to moderate in effect size, with the exception of everyday discrimination, which was associated with large decreases in self-rated health. The associations between concealment, internalized stigma, connectedness, and self-rated health were negligible (CIs spanning zero).

Model 2 (Table 3) presents tests of interactions examining the extent to which the associations between minority stress, community connectedness, and self-rated health differed by cohort. Similar to psychological distress, the interaction between felt stigma and cohort was appreciable (see Fig. 2), in which the negative association between stigma and self-rated health was more pronounced among members of the older cohorts (small to moderate association) than among members of the younger cohort (association near zero). Interactions between cohort and the other minority stressors and community connectedness were negligible (near zero effect sizes).

**Fig. 4:**
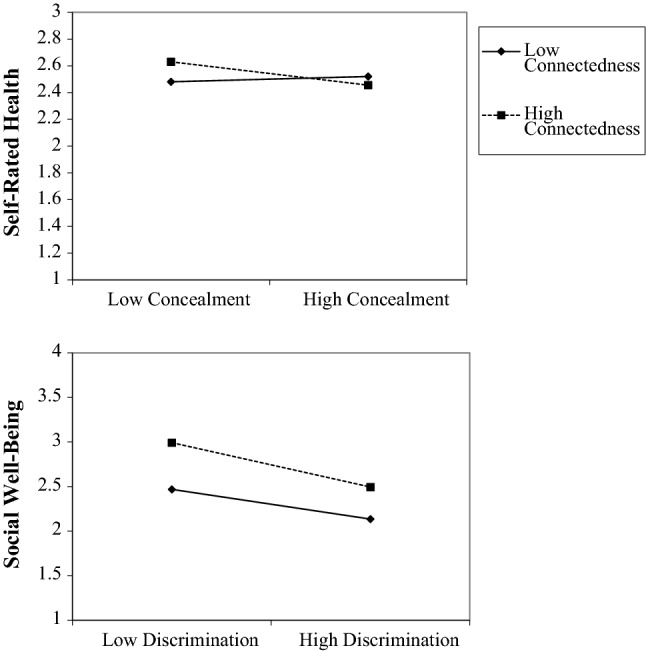
Community connectedness as a moderator of the relationship between minority stress and health outcomes

Model 3 (Table 3) presents tests of interactions examining the extent to which the associations between minority stressors and self-rated health varied by levels of community connectedness. We observed an interaction between stigma concealment and connectedness (see Fig. 4), wherein increased levels of stigma concealment were associated with poorer self-rated health for those with higher levels of community connectedness (small to moderate association), but not for those with lower levels of community connectedness (near zero association). Interactions between community connectedness and indicators of minority stress were negligible, with effect sizes approaching zero.

### Outcome 3: Social Well-Being

As shown in Table 4, Model 1 levels of social well-being demonstrated small declines over time, on average, for all participants, but differences in social well-being between the younger cohort and members of the older cohorts were negligible.

**Table 4:** Generalized estimating equations predicting social well-being based on minority stress and community connectedness across cohort and time

Parameter	Model 1: Main effects				Model 2: Cohort by Minority Stress Interactions				Model 3: Connectedness by Minority Stress Interactions			
	B	95% CI		*p*	B	95% CI		*p*	B	95% CI		*p*
		Lower	Upper			Lower	Upper			Lower	Upper	
Intercept	4.83	4.75	4.90	< 0.01	4.82	4.75	4.90	< 0.01	4.82	4.74	4.90	< 0.01
Black	− 0.04	− 0.15	0.07	0.50	− 0.04	− 0.15	0.07	0.47	− 0.04	− 0.16	0.07	0.44
Latino	− 0.05	− 0.15	0.05	0.29	− 0.06	− 0.16	0.04	0.24	− 0.05	− 0.15	0.05	0.30
Female sex	− 0.02	− 0.11	0.06	0.62	− 0.02	− 0.10	0.06	0.65	− 0.01	− 0.10	0.07	0.76
Non-binary gender	− 0.12	− 0.28	0.05	0.16	− 0.10	− 0.26	0.06	0.24	− 0.11	− 0.27	0.05	0.19
Plurisexual	− 0.10	− 0.20	0.00	0.05	− 0.11	− 0.21	− 0.01	0.04	− 0.11	− 0.21	− 0.01	0.04
High school or less	− 0.28	− 0.39	− 0.17	< 0.01	− 0.28	− 0.39	− 0.17	< 0.01	− 0.28	− 0.39	− 0.17	< 0.01
Younger cohort	− 0.03	− 0.12	0.07	0.60	− 0.02	− 0.11	0.07	0.62	− 0.02	− 0.12	0.07	0.66
Time	− 0.10	− 0.14	− 0.07	< 0.01	− 0.10	− 0.13	− 0.06	< 0.01	− 0.11	− 0.14	− 0.07	< 0.01
Victimization	− 0.01	− 0.06	0.04	0.75	0.02	− 0.04	0.08	0.43	− 0.01	− 0.06	0.04	0.67
Discrimination	− 0.31	− 0.38	− 0.25	< 0.01	− 0.32	− 0.41	− 0.23	< 0.01	− 0.31	− 0.38	− 0.25	< 0.01
Felt stigma	− 0.19	− 0.23	− 0.15	< 0.01	− 0.24	− 0.29	− 0.18	< 0.01	− 0.19	− 0.23	− 0.15	< 0.01
Concealment From Family	− 0.02	− 0.06	0.03	0.45	− 0.02	− 0.07	0.04	0.60	− 0.02	− 0.06	0.03	0.40
Internalized stigma	− 0.01	− 0.06	0.04	0.62	− 0.04	− 0.11	0.03	0.29	− 0.02	− 0.07	0.03	0.54
Connectedness	0.40	0.33	0.48	< 0.01	0.47	0.37	0.57	< 0.01	0.40	0.33	0.47	< 0.01
Younger * victimization					− 0.10	− 0.19	0.00	0.04				
Younger * discrimination					0.02	− 0.10	0.14	0.76				
Younger * stigma					0.12	0.04	0.19	< 0.01				
Younger * Concealment					0.01	− 0.07	0.08	0.87				
Younger * internalized					0.07	− 0.03	0.16	0.20				
Younger * connectedness					− 0.17	− 0.31	− 0.03	0.02				
Connectedness * victimization									0.07	− 0.01	0.15	0.07
Connectedness * Discrimination									− 0.11	− 0.22	− 0.01	0.04
Connectedness * Stigma									0.04	− 0.03	0.12	0.23
Connectedness * Concealment									− 0.05	− 0.11	0.02	0.17
Connectedness * Internalized									0.01	− 0.08	0.09	0.87
Scale/Link Parameter	0.63				0.63				0.63			

Regarding the associations between minority stressors and social well-being, higher levels of everyday discrimination and felt stigma were associated with small to moderate decreases in their levels of social well-being. However, the associations between victimization, stigma concealment, internalized stigma, and social well-being were negligible in magnitude. Higher levels of community connectedness were associated with increases in social well-being that were moderate to large in magnitude.

Model 2 (Table 4) presents tests of interactions examining the extent to which the associations between minority stressors, community connectedness, and social well-being varied by cohort. We again observed an interaction between felt stigma and cohort (see Fig. 2), in which the negative association between stigma and social well-being was appreciable for all cohorts, but stronger in magnitude for the older cohorts than the younger cohort. Interactions between cohort and the other minority stressors in predicting social well-being were negligible. However, similar to psychological distress, we observed an interaction between community connectedness and cohort (see Fig. 3), in which the association between community connectedness and social well-being was present for all cohorts, but stronger in magnitude for the older cohorts than the younger cohort.

Model 3 (Table 4) presents tests of interactions examining the extent to which the association between minority stress and social well-being varied by levels of community connectedness. We observed an interaction between everyday discrimination and connectedness in predicting social well-being (see Fig. 4), wherein the negative association between everyday discrimination and social well-being was present for all cohorts, but slightly stronger in magnitude for people with higher levels of connection to the community than among people with lower levels of connection to the community. Interactions between community connectedness and indicators of minority stress in predicting social well-being were negligible.

### Sensitivity Analyses

We conducted sensitivity analyses to examine whether the theoretically-based decision to combine the middle and older cohorts in comparisons with the younger cohort were statistically justified. Specifically, we re-ran models testing the interactions between cohort and minority stressors and connectedness in predicting the three health outcomes with interaction terms comparing younger to middle cohort members and younger to older cohort members. These supplemental analyses are presented in Table S1. Comparing the effect sizes and confidence intervals for the different cohort interaction effects indicated only slight differences, with similar effect sizes and largely overlapping confidence intervals. Given the lack of substantial statistical differences, combined with the conceptual justification provided earlier, we did not further explore differences in the associations between minority stress, connectedness, and health outcomes separating out the middle and older cohorts.

## Discussion

Drawing on data obtained from a national probability sample using a longitudinal design, we showed that minority stress remains an important factor associated with the health and well-being of sexual minority individuals, from both young and older cohorts. Despite the improving social and legal environment, younger sexual minority people continue to experience minority stress. Overall, multiple indicators of minority stress were associated with higher levels of psychological distress, poorer self-rated health, and poorer social well-being. Community connectedness was associated with lower levels of psychological distress and higher levels of social well-being. Supporting the hypotheses we posed, we found that the associations between more proximal (but not distal) forms of minority stress (i.e., felt stigma) and health were less pronounced in the younger cohort compared to older cohorts. We also found evidence that the salutogenic role of community connectedness was less pronounced for the younger cohort than among older cohorts. However, findings regarding the stress-buffering role of community connectedness were inconsistent.

Everyday discrimination and felt stigma were shown to have a negative association with all health and well-being outcomes measured in the study. Experiences of victimization also had a negative association with mental and physical health outcomes, although victimization was not associated with social well-being. Consistent with previous research, internalized stigma had a negative association with mental health (Newcomb & Mustanski, 2010), but was not meaningfully associated with physical health or social well-being.

As hypothesized, some of these negative associations between minority stress and health and well-being were diminished in the younger cohort compared with the older cohort of sexual minority individuals. This finding provides some evidence for the claim that the changing social climate has diminished the negative role of minority stress in shaping health and well-being. This pattern was specific to the effects of felt stigma but not the other forms of minority stress measured in the study. Namely, the negative association between experiences of felt sigma and health was less pronounced in the younger cohort than for the older cohorts of sexual minority individuals. That this pattern was specific to felt stigma may be related to the proximal nature of felt stigma as a minority stressor, which is grounded in individuals’ appraisals of their social environments as stigmatizing, manifesting in expectations of rejection and marginalization (Meyer, 2003). It is plausible that younger sexual minority people are benefiting from a peer group that is more accepting of them and are therefore less likely than older sexual minority people to expect rejection and discrimination. The association between felt stigma and health and well-being in the younger cohort may likely be diminished by the fact that sexual minority emerging adults have come of age in a more accepting and inclusive social environment compared to older cohorts (Meyer, 2016; Roberts, 2019).

In contrast, we did not observe any differences by cohort in the associations between the more distal minority stressors of victimization and discrimination and health, which stem more directly from the social environment and are perpetrated by other social actors (e.g., family, coworkers, strangers). These distal minority stressors are theorized to occur independent of individuals’ self-appraisals of their social environment as threatening (Frost & Meyer, 2015), which may explain why we did not observe any cohort differences in their associations with health and well-being.

We also found evidence for the health implications of feeling a psychological sense of connectedness to a community of other sexual minority individuals. In general, an increased sense of connectedness to an LGBT community was associated with improvements in social well-being and reductions in mental health symptoms in the form of psychological distress. But the positive associations of community connectedness did not extend to physical health.

The observed benefits of community connectedness for mental health and social well-being were appreciably less prominent in the younger cohort than they were in the older cohorts of sexual minority individuals. This may lend further support to the social change hypothesis, which suggests that because sexual minority emerging adults came of age in a social environment in which their peers generally were more accepting and held more positive attitudes about sexual diversity, they may not need affirmation from other sexual minority people as much as older sexual minority individuals needed.

Our findings regarding the positive association between community connectedness and mental health and social well-being are in line with previous research showing similar associations (Frost & Meyer, 2012; Scroggs & Vennum, 2021) and provide partial support for the hypothesized salutogenic effect of community connectedness. However, we did not find evidence for the theorized stress buffering role of community connectedness as a community coping resource. Instead, we found some evidence that an increased sense of community connectedness can magnify the negative association between discrimination and social well-being and stigma concealment and self-rated health. Research on community connectedness would benefit from further investigation regarding the temporal ordering of its associations with minority stress and health. For example, one explanation for the current study’s finding could be that people who experience minority stress and its potential negative health outcomes may be more likely to seek out support from communities of similar others. We are not able to interpret these effects further, given the small effect sizes and lack of consistency observed across the various stressors and health outcomes investigated in this study. Future theory and research on community connectedness can benefit from further clarifying the role of community connectedness in the minority stress experience (e.g., direct effect vs. stress buffer), potential additional variables at play in this association, and the degree to which community connectedness matters for the health of younger cohorts of sexual minorities.

The focus of the current study was on the associations between minority stress, community connectedness, and health and the degree to which they differed between the younger cohort of emerging adults and older cohorts who came of age in more accepting social climates. This focus is notably different from previous studies that have compared cohorts on mean exposure to minority stressors and community connectedness, which is a related but distinct research aim. As noted in the descriptive analyses presented in the current study, a lack of mean differences between cohorts in their experiences of minority stress and community connectedness are not indicative of the degree to which minority stress and community connectedness are related to health outcomes. For example, there were no appreciable differences between the younger and older cohorts on mean levels of felt stigma, but the negative association between felt stigma and health outcomes was greater for the older cohorts than it was for the younger cohort. Thus, the current study shows that researchers interested in the role of social change in the experience of minority stress will benefit from greater attention to the potentially changing nature of the *association* between minority stress and health, rather than limiting their foci to group mean differences across cohorts.

### Limitations

Our findings should be interpreted in light of several limitations. First, although the study utilized a longitudinal design, there are limits to the extent to which causal claims can be drawn from the data. The one-year interval between surveys allowed for sufficient change to be observed in the study variables; however, the length of time between assessments combined with the varied reporting period of outcomes and predictors limit our claims to contemporaneous effects. Thus, we cannot draw conclusions about the temporal ordering of some of the associations reported in this paper and we cannot rule out potential causal pathways that operate in the reverse direction (e.g., higher levels of psychological distress may lead people to perceive more minority stress).

Second, variables unmeasured by the current study may also influence participants’ self-reports of minority stress and health outcomes (e.g., personality traits). Relatedly, we are not able to attribute all experiences of minority stress as directly related to sexual minority identity. We did not use follow-up attribution items with reference to specific identities (e.g., race, gender, sexual orientation) for measures of victimization and discrimination to minimize attribution bias (e.g., it is not always possible to know why someone is behaving in discriminatory way) and so these experiences could be assessed in an intersectional way that would be applicable for all participants and suitable for between-group comparisons (following recommendations in Meyer et al., 2008).

Additional limitations center around the lower than ideal retention across the three waves of the survey (a comprehensive analysis of attrition across waves in the Generations Study is provided in Krueger et al., 2020). Of note, participants who were younger, less educated, of fair health, and those who identified as Black or LatinX were less likely to be retained across Waves. Although these patterns of attrition are noted in other longitudinal studies, they may nonetheless introduce bias into our analyses and future studies should implement mechanisms to retain participants from these important subgroups in the larger sexual minority population. Our approach to analysis using GEEs was aligned with our interest in group-level effects, but precluded the ability to examine intraindividual changes, which were outside the scope of the present investigation.

Finally, we have been careful to focus our claims on sexual minority individuals throughout this paper. Although gender nonbinary people who were not transgender were included in this study, transgender individuals were not included. This was because transgender individuals were recruited into a separate study that allowed for a focus on their unique concerns. The number of non-binary identified individuals was too small to permit sub-group specific analyses or to test as a moderator of the associations between our focal theoretical constructs and health outcomes. Future research should investigate the degree to which the associations and cohort differences observed in the present study generalize to gender minority individuals.

### Conclusions

Despite these limitations, the study has many strengths. Specifically, this is the first longitudinal national probability study of sexual minorities that includes questions that probe issues specific to the population, including minority stress and community connectedness. A further strength of the study is the use of three validated health outcomes reflecting different domains of health and well-being. The minority stress model (Meyer, 2003) explains the heightened incidence of mental health problems and disorders among sexual minorities. It has since been extended to explain a broader range of outcomes pertaining to physical health (Lick et al., 2013) and well-being (Kertzner et al., 2009). The model’s hypothesized explanation for health problems is social stigma, which should (in theory) exert a negative impact across multiple indicators of health, as opposed to having an isolated impact on a given specific domain (e.g., mental health) or disorder (e.g., generalized anxiety disorder). The present focus on the associations between minority stress and three indicators and domains of health, rather than specific domains or disorders in isolation, enhances the rigor of the investigation and provides a robust test of the core premises of the model.

This study focused on age cohort differences in the sexual minority population and provides valuable information about variability in sexual minority populations regarding social determinants of health, including both risk and protective factors, unique to the experiences of sexual minority individuals (Schwartz & Meyer, 2010). Our results show that despite improvements in the social environment of sexual minority people in the USA, they continue to experience the negative association between minority stress and health and well-being. It further showcases the importance of attention to variability across the life course (Hammack et al., 2018) in the applicability of the minority stress model, which has become perhaps the most influential framework guiding public health research and interventions aimed at understanding and addressing health inequalities faced by sexual minority populations (Chaudoir et al., 2017; Hatzenbuehler & Pachankis, 2016; National Academies of Sciences, Engineering, & Medicine, 2020). The attention devoted to positive social and policy change in the popular media should not distract from the continuing negative association between minority stress and the health and well-being of sexual minorities (Meyer, 2016), which although sometimes slightly attenuated, persists even for young cohorts of sexual minority emerging adults who came of age in more accepting social climates.

## Supplementary Information

Below is the link to the electronic supplementary material.

Supplementary file1 (PDF 43 kb)

## Declarations

### Availability of Data and Material

These data are publicly available via the Inter-university Consortium for Political and Social Research (ICPSR) website at: https://www.icpsr.umich.edu/web/DSDR/studies/37166

### Code Availability

The final code used for the analyses reported in the published version of the manuscript will be made available upon request by emailing the corresponding author.
