# Supplementary material for: Social Change and the Health of Sexual Minority Individuals: Do the Effects of Minority Stress and Community Connectedness Vary by Age Cohort?
Source: Arch Sex Behav. 2022 Apr 11;51(4):2299–316. doi: 10.1007/s10508-022-02288-6 (PMC9192485; doi:10.1007/s10508-022-02288-6)
Supplement: Supplementary file 1 — Supplementary file1 (PDF 43 kb) [file 10508_2022_2288_MOESM1_ESM.pdf]

Table S1. Sensitivity Analyses Examining Interactions for Middle vs. Younger and Older vs. Younger Cohorts.

	Psychological Distress				Self-Rated Health				Social Well-Being			
		95% CI				95% CI				95% CI		
Parameter	B	Lower	Upper	p	B	Lower	Upper	p	B	Lower	Upper	p
Intercept	7.75	7.16	8.34	0.00	2.86	2.75	2.97	0.00	4.80	4.69	4.90	0.00
Black	-0.94	-1.59	-0.29	0.00	0.04	-0.10	0.17	0.60	-0.04	-0.15	0.08	0.54
Latino	-0.04	-0.59	0.52	0.90	-0.09	-0.21	0.03	0.14	-0.05	-0.15	0.05	0.30
Female Sex	0.22	-0.22	0.66	0.33	-0.12	-0.22	-0.02	0.02	-0.02	-0.11	0.06	0.60
Non-Binary Gender	1.15	0.20	2.10	0.02	-0.16	-0.36	0.04	0.12	-0.10	-0.26	0.06	0.22
Plurisexual	1.17	0.62	1.73	0.00	-0.18	-0.30	-0.07	0.00	-0.10	-0.20	0.00	0.05
Highschool or Less	0.51	-0.07	1.09	0.09	-0.16	-0.28	-0.04	0.01	-0.28	-0.38	-0.17	0.00
Middle Cohort (=1)	-1.81	-2.37	-1.24	0.00	-0.21	-0.33	-0.09	0.00	-0.01	-0.11	0.09	0.87
Older Cohort (=1)	-2.59	-3.21	-1.97	0.00	-0.42	-0.55	-0.28	0.00	0.06	-0.06	0.18	0.34
Time	0.31	0.13	0.50	0.00	-0.07	-0.11	-0.03	0.00	-0.10	-0.13	-0.06	0.00
Victimization	0.82	0.33	1.31	0.00	-0.11	-0.20	-0.01	0.03	-0.07	-0.15	0.00	0.06
Discrimination	2.58	2.06	3.10	0.00	-0.30	-0.40	-0.20	0.00	-0.30	-0.39	-0.21	0.00
Felt Stigma	0.09	-0.24	0.42	0.59	-0.09	-0.15	-0.02	0.01	-0.12	-0.18	-0.07	0.00
Concealment From Family	0.19	-0.14	0.53	0.26	-0.05	-0.11	0.02	0.16	-0.01	-0.07	0.05	0.73
Internalized Stigma	0.44	-0.01	0.89	0.05	-0.03	-0.11	0.06	0.54	0.03	-0.04	0.09	0.46
Connectedness	0.07	-0.53	0.68	0.82	0.03	-0.08	0.14	0.58	0.30	0.20	0.41	0.00
Middle * Victimization	-0.12	-0.78	0.54	0.72	0.05	-0.08	0.19	0.44	0.08	-0.03	0.19	0.16
Older * Victimization	-0.27	-0.87	0.33	0.37	0.01	-0.12	0.14	0.85	0.11	0.00	0.21	0.04
Middle * Discrimination	0.25	-0.57	1.07	0.55	-0.01	-0.18	0.15	0.90	-0.01	-0.15	0.13	0.89
Older * Discrimination	-0.20	-1.01	0.61	0.63	-0.10	-0.29	0.09	0.30	-0.01	-0.18	0.16	0.91
Middle * Stigma	0.57	-0.02	1.15	0.06	-0.12	-0.23	0.00	0.05	-0.07	-0.17	0.03	0.17
Older * Stigma	0.69	0.17	1.21	0.01	-0.11	-0.23	0.01	0.06	-0.16	-0.25	-0.06	0.00
Middle * Concealment	-0.38	-0.88	0.12	0.14	0.03	-0.07	0.14	0.56	0.00	-0.09	0.09	0.99
Older * Concealment	0.02	-0.50	0.53	0.95	0.04	-0.07	0.15	0.50	0.00	-0.10	0.10	0.94
Middle * Internalized	0.12	-0.56	0.80	0.73	0.05	-0.10	0.19	0.53	-0.11	-0.23	0.01	0.08
Older * Internalized	-0.33	-1.00	0.34	0.33	-0.08	-0.21	0.06	0.27	-0.03	-0.15	0.09	0.63
Middle * Connectedness	-1.23	-2.18	-0.27	0.01	0.15	-0.05	0.35	0.14	0.17	-0.01	0.35	0.07
Older * Connectedness	-0.63	-1.46	0.19	0.13	-0.07	-0.25	0.11	0.47	0.17	0.01	0.33	0.04
Scale/Link Parameter	18.38				0.85				0.63			
